# Causes and Five-Year Proportion of New Irreversible Visual Impairment in Jinshan District, Shanghai, from 2009–2018

**DOI:** 10.1155/2021/8873283

**Published:** 2021-07-28

**Authors:** Tao Li, Xiaodong Zhou

**Affiliations:** ^1^Department of Ophthalmology, Jinshan Hospital of Fudan University, Shanghai, China; ^2^Department of Ophthalmology, Eye & ENT Hospital of Fudan University, Shanghai, China

## Abstract

**Purpose:**

To describe the age distribution and main causes of new registered irreversible visual impairment (VI) and to compare the five-year proportion of VI in Jinshan district, Shanghai, from 2009 to 2018.

**Methods:**

The new irreversible VI data were collected in the registry system from the Disabled Persons' Federation in Jinshan district from January 1, 2009, to December 31, 2018. Age, gender, and causes of VI were included, and the 5-year proportion of VI was calculated.

**Results:**

The peak occurrence of blindness occurred in the 50–59 yrs group in 2009–2013 and in the ≥70 yrs group in 2014–2018. The peak occurrence of low vision occurred in the 40–49 yrs group in 2009–2013 and in the 50–59 yrs group in 2014–2018. Myopic macular degeneration (MMD, 15.5%), diabetic retinopathy (DR, 14.3%), and other optic nerve atrophy (ONA, 14.3%) were the three leading causes of blindness in 2009-2013, whereas MMD (21.3%), age-related macular degeneration (AMD, 19.6%), ONA (14.9%) were the three leading causes of blindness in 2014–2018. MMD (39.2%), DR (9.6%), ONA (8.8%) were the three leading causes of low vision in 2009–2013, whereas MMD (38.7%), AMD (23.3%), ONA (7.4%) were the three leading causes of low vision in 2014–2018. The proportions of blindness and low vision caused by AMD were higher in 2014–2018 than those in 2009–2013 (*P*=0.034 and *P* < 0.001, respectively).

**Conclusion:**

The present study demonstrated an increasing trend in the number of irreversibly visually impaired individuals from 2009 to 2018. More attention should be paid to people with high myopia and old age.

## 1. Introduction

Irreversible visual impairment (VI), including blindness and low vision, is a major public health problem, affecting more than 250 million people worldwide [1]. The number of irreversible VI is deemed to accumulate with the aging population. In China, the proportion of population aged 60 yrs and older is reported to rise from 13.3% in 2010 to 34% in 2050 [2], and there are 12.63 million individuals with irreversible VI in 2010 [3] and approximately 6.02 million individuals with blindness in 2015 [4]. Irreversible VI can lead to significant disease burden and correlate with poverty [5], and reduce the quality of life of affected individuals [6]. So, estimating the trend of new irreversible VI is important for adjusting intervention strategies in China, especially in rural areas with lack of medical resources.

Jinshan district is located at southwestern section of Shanghai city, China. It has a population of more than 730,000. The Disabled Persons' Federation is a unified organization of all kinds of disabled persons in China. Its main responsibility is to investigate the status of the disabled, count their data, and analyze their causes. In addition, it can carry out technical services in rehabilitation and prevention for the disabled and carry out activities to effectively improve the production and living conditions of the disabled. Registry data can provide updated information on the trends of the age distribution and causes of the affected individuals. The purpose of this study was to describe the age distribution and main causes of new registered irreversible VI and to compare the five-year proportion of VI in Jinshan district from 2009 to 2018.

## 2. Methods

This study was approved by the Ethics Committee of Jinshan Hospital of Fudan University, Shanghai. All study procedures adhered to the tenets of the Declaration of Helsinki. Written informed consent was obtained from all the affected individuals.

The new irreversible visual impairment (VI) data were retrospectively collected in the registry system from the Disabled Persons' Federation in Jinshan district from January 1, 2009, to December 31, 2018. According to the World Health Organization (WHO) definition, blindness is defined as best-corrected visual acuity (BCVA) worse than 20/400 (3/60) in the better-seeing eye, or a corresponding visual field loss to less than 10 degrees in the better-seeing eye; low vision is defined as BCVA worse than 20/60 (6/18) but better than 20/400 (3/60) in the better-seeing eye. Persons who did not meet the criteria of VI were excluded. There were 956 persons with VI, including 380 blind persons and 576 low vision persons. Age, gender, and causes of VI were included, and the 5-year proportion of VI was calculated.

A comprehensive ocular examination was performed on every applicant. Uncorrected visual acuity (UCVA) was measured with a standardized visual acuity chart (Shenguang; Yuejin Medical Optical Device Factory, Shanghai, China). Subject refraction was measured with a synthetic optometry (CV-5000; Topcon Corporation, Tokyo, Japan). Intraocular pressure was measured with a noncontact tonometer (CT-800; Topcon Corporation, Tokyo, Japan). Slit lamp (DC-4; Topcon Corporation, Tokyo, Japan) was used to examine the anterior segment of the eyes. Color fundus photography (Visucam 200; Carl Zeiss Meditec, Inc., Dublin, USA) and optical coherence tomography (Cirrus-HD OCT 4000; Carl Zeiss Meditec, Inc., Dublin, USA) were used to examine the posterior segment of the eyes. Visual field tests (BIO-1000, Beiao Electronic Instrument Co., Ltd., Chongqing, China) were performed in the applicants with glaucoma, retinitis pigmentosa (RP), and other optic nerve atrophy (ONA).

The causes of blindness and low vision were classified according to the International Classification of Diseases, Tenth Revision, Clinical Modification (ICD-10-CM). All cases were assigned a principal cause of blindness and low vision by the examining ophthalmologist using a 13-item list (genetic, congenital, or developmental disorder, cataract, glaucoma, trachoma, corneal disease, optic lesion, retinal and pigmented membrane lesions, refractive error, amblyopia, ocular trauma, toxicosis, other causes, and undetermined causes). Furthermore, retinal and pigmented membrane lesions were divided to 5 categories: age-related macular degeneration (AMD), diabetic retinopathy (DR), RP, retinal detachment, and others. The list of refractive error included the myopic macular degeneration (MMD) with one or more of the following ophthalmologic findings: tessellated fundus, diffuse or patchy chorioretinal atrophy, macular atrophy, lacquer cracks, choroidal neovascularization (CNV), Fuchs spot, or posterior staphyloma.

The disease leading to the greatest vision loss was identified as the principal cause of blindness and low vision. The causes of blindness and low vision in both eyes were recorded, but only data from the better-seeing eye were used for analysis. If cataract was identified as the principal cause of blindness and low vision, the individual was advised for surgery and reassessed at least 2 months postoperatively if BCVA was still worse than 20/60 (6/18).

Statistical analysis was performed using SPSS software version 17 (SPSS, Inc., Chicago, IL). Age was grouped as ≤29 yrs, 30–39 yrs, 40–49 yrs, 50–59 yrs, 60–69 yrs, and ≥70 yrs. Independent *t*-test was used to compare the difference of age between 2009–2013 and 2014–2018. Chi-square test was used to analyze the differences in the age distribution and proportion of blindness and low vision between 2009–2013 and 2014–2018. *P* < 0.05 was considered as statistically significant.

## 3. Results

**Figure 1:**
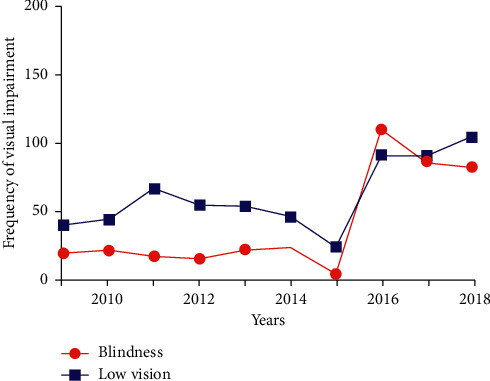
Frequency of visual impairment from 2009–2018.

As shown in Figure 1, from 2009 to 2018, the numbers of the new blindness were 17, 19, 15, 13, 20, 21, 2, 109, 85, and 81, respectively, and the numbers of the new low vision were 38, 42, 65, 53, 52, 44, 22, 90, 89, and 103, respectively. The overall average age was 56.2 ± 14.3 yrs, and the average age increased from 50.0 ± 11.9 yrs in 2009–2013 to 59.5 ± 14.4 yrs in 2014–2018 (*P* < 0.001).

**Figure 2:**
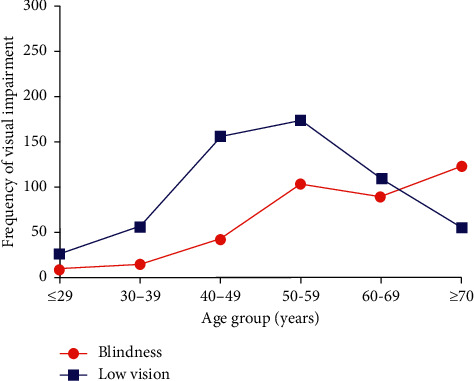
Frequency of visual impairment in different age groups.

Table 1 shows the age and gender distribution of blindness and low vision in Jinshan district from 2009 to 2018. The peak occurrence of blindness occurred in the 50–59 yrs group in 2009–2013 and in the ≥70 yrs group in 2014–2018. The proportion of blindness in the ≥70 yrs group was higher in 2014–2018 than that in 2009–2013 (*P* < 0.001), whereas the proportion of blindness in the 40–49 yrs group was lower in 2014–2018 than that in 2009–2013 (*P*=0.005). The peak occurrence of low vision occurred in the 40–49 yrs group in 2009–2013 and in the 50–59 yrs group in 2014–2018. The proportions of low vision in the 60–69 yrs and ≥70 yrs groups were higher in 2014–2018 than those in 2009–2013 (both *P* < 0.001), whereas the proportions of low vision in the 30–39 yrs and 40–49 yrs groups were lower in 2014–2018 than those in 2009–2013 (*P*=0.016 and *P* < 0.001, respectively).  Figure 2 shows the peak occurrence of blindness occurred in the ≥70 yrs group, whereas the peak occurrence of low vision occurred in the 50–59 yrs group in 2009–2018.

The leading causes of blindness and low vision in 2009–2018 are summarized in Table 2. The three leading causes of blindness were MMD (20.0%), AMD (17.4%), and ONA (14.7%). Furthermore, MMD (15.5%), DR (14.3%), and ONA (14.3%) were the three leading causes of blindness in 2009–2013, whereas MMD (21.3%), AMD (19.6%), and ONA (14.9%) were the three leading causes of blindness in 2014–2018. The three leading causes of low vision were MMD (38.9%), AMD (17.4%), and ONA (7.8%). Furthermore, MMD (39.2%), DR (9.6%), and ONA (8.8%) were the three leading causes of low vision in 2009–2013, whereas MMD (38.7%), AMD (23.3%), and ONA (7.4%) were the three leading causes of low vision in 2014–2018. The proportions of blindness and low vision caused by AMD were higher in 2014–2018 than those in 2009–2013 (*P*=0.034 and *P* < 0.001, respectively). However, the proportion of low vision in the other group was lower in 2014–2018 than that in 2009–2013 (*P*=0.003).

## 4. Discussion

Bourne et al. [1] reported that the estimated number of people with blindness and low vision increased from 30.6 million and 159.9 million in 1990 to 36.0 million and 216.6 million in 2015, respectively, which may be due to population growth, population aging, and reduction in age-specific prevalence. With China entering an aging society, understanding the information of new irreversible VI will help the government formulate better health service policies, especially in rural areas. Thus, we analyzed new irreversible VI data based on the registry system in Jinshan district, a rural district of Shanghai.

In the study, the peak age distribution of blindness was greater than that of low vision from 2009 to 2018. The proportion of blindness in the ≥70 yrs group and the proportions of low vision in the 60–69 yrs and ≥70 yrs groups were higher in 2014–2018 than those in 2009–2013. These meant that the peak age distribution increased in 2014–2018 compared to 2009–2013. This may be due to the aging population in China and the fact that individuals with low vision became blind with the increase of age. In Turkey, the most frequently occurring age group of blind people was the 80–89 yrs group with cataract as the principal cause [7]. In addition, the peak occurrence of blindness occurred in the 50–59 yrs group in 2009–2013 and in the ≥70 yrs group in 2014–2018, whereas the peak occurrence of low vision occurred in the 40–49 yrs group in 2009–2013 and in the 50–59 yrs group in 2014–2018. These suggested that blind individuals were older and low vision mainly affected the working age individuals.

In this study, MMD was the most common cause of blindness from 2009 to 2018, without significant difference in the proportion between 2009—2013 and 2014–2018, which was consistent with the findings of Jing'an district, Shanghai [8, 9]. The first leading cause of new blindness was MMD in Jing'an district, Shanghai, from 2001 to 2015 [8, 9]. In Netherlands, MMD was the main cause of impaired vision among people younger than 75 yrs. [10]. However, the result was different from other studies. MMD was the second leading cause of blindness in Beijing [11] and Guangzhou [12]. In some countries, MMD was responsible for a small number of blind individuals [13, 14]. These differences may be due to socioeconomic development levels, study methods, and the criteria of blindness, and so on. If some treatable diseases (e.g., cataract and uncorrected refractive error) were excluded as causes of VI in these studies, MMD would be a major cause of blindness.

In the present study, AMD was the fourth leading cause of blindness (9.5%) in 2009–2013 and rose to the second leading cause of blindness (19.6%) in 2014–2018. AMD with abnormal CNV under or nearby the macula or not affected a great number of the aging population worldwide [15, 16] and was one of the leading causes of blindness in the industrialized countries [17, 18]. Our findings were similar to those in Jing'an district, Shanghai, from 2001 to 2015, suggesting that AMD was the third leading cause of blindness [8, 9]. AMD was the major cause of blindness among the white population [19] and white Americans (54%) [20] and in Scandinavia (42.8%) [13] and was the second leading cause of blindness in Turkey (21%) [7]. In Australia, AMD was the major cause of severe VI or blindness, affecting 0.45% of the population [21]. However, AMD was responsible for a few blind individuals (2.0%) in Beijing [11]. The proportion of blindness caused by AMD was higher in high-income regions with older populations due to the increasing risk of AMD with age [22]. With China's economic development, socioeconomic level and lifestyle have been changed in Shanghai, and the prevalence of AMD has increased.

In this study, ONA was the second leading cause of blindness from 2009 to 2018. In Jing'an district, ONA was the fourth leading cause of blindness in 2001–2003, the fifth leading cause in 2004–2006, the eighth leading cause in 2007–2009, the fifth leading cause in 2010–2012, and the sixth leading cause in 2013–2015 [8, 9]. ONA was one of the major causes of blindness worldwide [23]. Iwase et al. [24] found that the major cause of blindness was optic atrophy in Japan. Blindness due to ONA was the third leading cause, accounting for 11% of blind individuals in Barbados [25]. ONA was the major cause of VI in children and young adults in Poland [26]. A large variation in the prevalence of ONA among regions is responsible for this difference.

In the study, glaucoma was the third leading cause of blindness in 2009–2013 and fell to the fifth leading cause of blindness in 2014–2018. These were different from the findings of Jing'an district, Shanghai: glaucoma was the second leading cause of new blindness in Jing'an district, Shanghai, from 2001 to 2015 [8, 9]. The proportion of blindness caused by glaucoma varied notably among different regions, with the highest in Latin America [22]. This may be partly because more glaucoma patients received timely treatment and good control, or partly because affected individuals with other causes of blindness increased more and reduced the proportion of glaucoma in the study.

In this study, MMD and AMD were the top two causes of low vision from 2009 to 2018, which was consistent with the finding with Xia et al. [9]: the leading causes of low vision were MMD and AMD in Jing'an district, Shanghai, from 2010 to 2015. In this study, there were no significant differences in the prevalence of MMD as the low vision cause between 2009–2013 and 2014–2018. High myopia-related macular degeneration was becoming the major cause of low vision in some Asian countries [8, 24, 27]. In addition, the proportion of AMD as the low vision cause was increased from 9.6% in 2009–2013 to 23.3% in 2014–2018. Similar to the blindness, economic level has important influence on the proportion of AMD in the low vision population. With the development of economy in Shanghai, individuals with AMD will increase.

There are some limitations in our study. Firstly, only new irreversible VI was analyzed in this study, whereas avoidable VI due to preventable or treatable causes (e.g., cataract and uncorrected refractive error) was not included. Secondly, our data only showed the proportion of blindness and low vision in Jinshan district, which could not represent the information of the whole Shanghai. However, this study could be mainly useful to determine the health service priorities for China government, especially in rural regions.

## 5. Conclusions

The present study demonstrates an increasing trend in the number of irreversibly visually impaired individuals from 2009 to 2018. The three leading causes of blindness and low vision are MMD, AMD, and ONA in 2009–2018. Thus, limited changes in the estimates of proportion of blindness and low vision suggest that more attention should be paid to people with high myopia and old age.

## Figures and Tables

**Table 1:** Age and gender distribution of visual impairment in Jinshan district, Shanghai, using the visual impairment definitions of the WHO.

	2009–2013				2014–2018					
	Blindness		Low vision		Blindness		Low vision			
Age group (yrs)	*n*	% (95% CI)	*n*	% (95% CI)	*n*	% (95% CI)	*n*	% (95% CI)	*P* value^a^	*P* value^b^
≤29	4	4.8 (0.1, 9.4)	12	4.8 (2.1, 7.5)	5	1.7 (0.2, 3.2)	14	4.3 (2.1, 6.5)	0.113	0.841
30–39	5	6.0 (0.8, 11.1)	33	13.2 (9.0, 17.4)	9	3.0 (1.1, 5.0)	23	7.1 (4.3, 9.8)	0.204	0.016
40–49	17	17.7 (9.9, 25.5)	100	40.0 (33.9, 46.1)	25	8.4 (5.3, 11.6)	56	17.2 (13.1, 21.3)	0.005	<0.001
50–59	30	35.7 (25.3, 46.2)	71	28.4 (22.8, 34.0)	73	24.7 (19.7, 29.6)	103	31.6 (26.5, 36.7)	0.052	0.412
60–69	16	19.0 (10.5, 27.6)	30	12.0 (7.9, 16.1)	73	24.7 (19.7, 29.6)	79	24.2 (19.6, 28.9)	0.310	<0.001
≥70	12	14.3 (6.6, 21.9)	4	1.6 (0, 3.2)	111	37.5 (32.0, 43.0)	51	15.6 (11.7, 19.6)	<0.001	<0.001
Male	49	58.3 (47.6, 69.1)	140	56.0 (49.8, 62.2)	158	53.4 (47.7, 59.1)	175	53.7 (48.2, 59.1)	0.458	0.613
Female	35	41.7 (30.9, 52.4)	110	44.0 (37.8, 50.2)	138	46.6 (40.9, 52.3)	151	46.3 (40.9, 51.8)	0.458	0.613
Total	84		250		296		326			

^a^Comparison of the prevalence of blindness between 2009–2013 and 2014–2018. ^b^Comparison of the prevalence of low vision between 2009–2013 and 2014–2018.

**Table 2:** Primary causes of visual impairment in the better-seeing eye in Jinshan district, Shanghai.

	2009–2013				2014–2018					
	Blindness		Low vision		Blindness		Low vision					
Causes	*n*	% (95% CI)	*n*	% (95% CI)	*n*	% (95% CI)	*n*	% (95% CI)	*P* value^a^	*P* value^b^

MMD	13	15.5 (7.6, 23.4)	98	39.2 (33.1, 45.3)	63	21.3 (16.6, 26.0)	126	38.7 (33.3, 44.0)	0.281	0.931
AMD	8	9.5 (3.1, 15.9)	24	9.6 (5.9, 13.3)	58	19.6 (15.0, 24.1)	76	23.3 (18.7, 27.9)	0.034	<0.001
DR	12	14.3 (6.6, 21.9)	12	4.8 (2.1, 7.5)	25	8.4 (5.3, 11.6)	24	7.4 (4.5, 10.2)	0.142	0.228
ONA	12	14.3 (6.6, 21.9)	22	8.8 (5.3, 12.3)	44	14.9 (10.8, 18.9)	23	7.1 (4.3, 9.8)	1.000	0.439
Glaucoma	11	13.1 (5.7, 20.5)	14	5.6 (2.7, 8.5)	22	7.4 (4.4, 10.4)	10	3.1 (1.2, 4.9)	0.123	0.145
RP	3	3.6 (−0.5, 7.6)	11	4.4 (1.8, 7.0)	20	6.8 (3.9, 9.6)	11	3.4 (1.4, 5.3)	0.436	0.521
Others	25	29.8 (19.8, 39.7)	69	27.6 (22.0, 33.2)	64	21.6 (16.9, 26.3)	56	17.2 (13.1, 21.3)	0.144	0.003

^a^Comparison of the prevalence of blindness between 2009–2013 and 2014–2018. ^b^Comparison of the prevalence of low vision between 2009–2013 and 2014–2018.

## Data Availability

The data used to support the findings of this study are available from the corresponding author upon request.

## References

[1] Bourne R., Flaxman S. R., Braithwaite T. (2017). Magnitude, temporal trends, and projections of the global prevalence of blindness and distance and near vision impairment: a systematic review and meta-analysis. *The Lancet Global Health*.

[2] Tabulation on the 2010 Population Census of the People’s Republic of China, 2012, http://www.stats.gov.cn/tjsj/pcsj/rkpc/6rp/indexch.htm

[3] The Total Number of Disabilities at the End of 2010, 2012, https://www.cdpf.org.cn/zwgk/zccx/cjrgk/4c0d47abe6a3414790d4ee786553fb65.htm

[4] Cheng C. Y., Wang N., Wong T. Y. (2020). Prevalence and causes of vision loss in East Asia in 2015: magnitude, temporal trends and projections. *The British Journal of Ophthalmology*.

[5] Polack S., Kuper H., Wadud Z. (2008). Quality of life and visual impairment from cataract in Satkhira district, Bangladesh. *The British Journal of Ophthalmology*.

[6] Wang W., Yan W., Muller A. (2017). Association of socioeconomics with prevalence of visual impairment and blindness. *JAMA Ophthalmology*.

[7] Mirza E., Mirza G. D., Oltulu R. (2019). The frequency and causes of blindness in a rural region of central anatolia of Turkey. *The Eurasian Journal of Medicine*.

[8] Wu L., Sun X., Zhou X., Weng C. (2011). Causes and 3-year-incidence of blindness in jing-an district, Shanghai, China 2001–2009. *BMC Ophthalmology*.

[9] Xia F., Wu L., Weng C., Zhou X. (2017). Causes and three-year incidence of irreversible visual impairment in Jing-an district, Shanghai, China from 2010–2015. *BMC Ophthalmology*.

[10] Klaver C. C. W., Wolfs R. C., Vingerling J. R. (1998). Age-specific prevalence and causes of blindness and visual impairment in an older population. *Archives of Ophthalmology*.

[11] Xu L., Wang Y., Li Y. (2006). Causes of blindness and visual impairment in urban and rural areas in Beijing: the Beijing Eye Study. *Ophthalmology*.

[12] Wang L., Huang W., He M. (2013). Causes and five-year incidence of blindness and visual impairment in urban Southern China: the Liwan Eye Study. *Investigative Ophthalmology & Visual Science*.

[13] Buch H., Vinding T., La Cour M., Appleyard M., Jensen G. B., Vesti Nielsen N. (2004). Prevalence and causes of visual impairment and blindness among 9980 Scandinavian adults. *Ophthalmology*.

[14] Sainz-Gómez C., Fernández-Robredo P., Salinas-Alamán Á. (2010). Prevalence and causes of bilateral blindness and visual impairment among institutionalized elderly people in Pamplona, Spain. *European Journal of Ophthalmology*.

[15] Ferris F. R., Wilkinson C. P., Bird A. (2013). Clinical classification of age-related macular degeneration. *Ophthalmology*.

[16] Campbell M., Doyle S. L. (2019). Current perspectives on established and novel therapies for pathological neovascularization in retinal disease. *Biochemical Pharmacology*.

[17] Velez-Montoya R., Oliver S. C. N., Olson J. L., Fine S. L., Quiroz-Mercado H., Mandava N. (2014). Current knowledge and trends in age-related macular degeneration. *Retina*.

[18] Daniel E., Pan W., Ying G. S. (2018). Development and course of scars in the comparison of age-related macular degeneration treatments trials. *Ophthalmology*.

[19] Figueroa A. G., McKay B. S. (2020). A G-protein coupled receptor and macular degeneration. *Cells*.

[20] Congdon N., O’Colmain B., Klaver C. C. (2004). Causes and prevalence of visual impairment among adults in the United States. *Archives of Ophthalmology*.

[21] Taylor H. R., Keeffe J. E., Vu H. T. (2005). Vision loss in Australia. *The Medical Journal of Australia*.

[22] Bourne R. R., Stevens G. A., White R. A. (2013). Causes of vision loss worldwide, 1990–2010: a systematic analysis. *The Lancet Global Health*.

[23] Chun B. Y., Rizzo J. R., White R. A. (2016). Dominant optic atrophy: updates on the pathophysiology and clinical manifestations of the optic atrophy 1 mutation. *Current Opinion in Ophthalmology*.

[24] Iwase A., Araie M., Tomidokoro A., Yamamoto T., Shimizu H., Kitazawa Y. (2006). Prevalence and causes of low vision and blindness in a Japanese adult population. *Ophthalmology*.

[25] Hyman L., Wu S. Y., Connell A. M. (2001). Prevalence and causes of visual impairment in the Barbados Eye Study. *Ophthalmology*.

[26] Kepa B., Hautz W., Seroczynska M., Adach K. (2007). Optic nerve atrophy--the main cause of visual impairment in children and young adults. *Medycyna Wieku Rozwojowego*.

[27] Varma R., Kim J. S., Burkemper B. S. (2016). Prevalence and causes of visual impairment and blindness in Chinese American adults. *JAMA Ophthalmology*.

